# Epcam, CD44, and CD49f Distinguish Sphere-Forming Human Prostate Basal Cells from a Subpopulation with Predominant Tubule Initiation Capability

**DOI:** 10.1371/journal.pone.0034219

**Published:** 2012-04-13

**Authors:** Changyong Guo, Haibo Liu, Bao-Hui Zhang, Radu M. Cadaneanu, Aqila M. Mayle, Isla P. Garraway

**Affiliations:** 1 Department of Urology, David Geffen School of Medicine at University of California Los Angeles, Los Angeles, California, United States of America; 2 Jonsson Comprehensive Cancer Center, David Geffen School of Medicine at University of California Los Angeles, Los Angeles, California, United States of America; 3 Greater Los Angeles Veterans Affairs Medical Center, Los Angeles, California, United States of America; Universitätsklinikum Carl Gustav Carus an der Technischen Universität Dresden, Germany

## Abstract

**Background:**

Human prostate basal cells expressing alpha-6 integrin (CD49f^Hi^) and/or CD44 form prostaspheres in vitro. This functional trait is often correlated with stem/progenitor (S/P) activity, including the ability to self-renew and induce differentiated tubules in vivo. Antigenic profiles that distinguish tubule-initiating prostate stem cells (SCs) from progenitor cells (PCs) and mature luminal cells (LCs) with less regenerative potential are unknown.

**Methodology/Principle Findings:**

Prostasphere assays and RT-PCR analysis was performed following FACS separation of total benign prostate cells based upon combinations of Epcam, CD44, and/or CD49f expression. Epithelial cell fractions were isolated, including Epcam^+^CD44^+^ and Epcam+CD44+CD49f^Hi^ basal cells that formed abundant spheres. When non-sphere-forming Epcam^+^CD44^−^ cells were fractionated based upon CD49f expression, a distinct subpopulation (Epcam^+^CD44^−^CD49f^Hi^) was identified that possessed a basal profile similar to Epcam^+^CD44^+^CD49f^Hi^ sphere-forming cells (p63^+^AR^Lo^PSA^−^). Evaluation of tubule induction capability of fractionated cells was performed, in vivo, via a fully humanized prostate tissue regeneration assay. Non-sphere-forming Epcam^+^CD44^−^ cells induced significantly more prostate tubular structures than Epcam^+^CD44^+^ sphere-forming cells. Further fractionation based upon CD49f co-expression identified Epcam^+^CD44^−^CD49f^Hi^ (non-sphere-forming) basal cells with significantly increased tubule induction activity compared to Epcam^+^CD44^−^CD49f^Lo^ (true) luminal cells.

**Conclusions/Significance:**

Our data delineates antigenic profiles that functionally distinguish human prostate epithelial subpopulations, including putative SCs that display superior tubule initiation capability and induce differentiated ductal/acini structures, sphere-forming PCs with relatively decreased tubule initiation activity, and terminally differentiated LCs that lack both sphere–forming and tubule-initiation activity. The results clearly demonstrate that sphere-forming ability is not predictive of tubule-initiation activity. The subpopulations identified are of interest because they may play distinct roles as cells of origin in the development of prostatic diseases, including cancer.

## Introduction

Human adult prostate S/Ps are characterized by surface marker expression, as well as functional traits, including the ability to self-renewal and differentiate into multiple lineages [1], [2], [3], [4], [5]. Markers that have been utilized to isolate human prostate S/Ps include Trop2, CD44, alpha_2_beta_1_-integrin^Hi^, alpha_6_-integrin^Hi^ (CD49f), and CD133 [1], [2], [4], [6]. However, a consensus does not exist regarding the antigenic profile of a functionally pure human prostate SC population and how to distinguish multipotent tubule-initiating SCs from progenitors with more limited potential. Making such a distinction may have important implications in understanding the etiology of prostatic disease, including benign prostatic hypertrophy and cancer.

Sphere-forming cells isolated from dissociated primary tissues are enriched in S/P cells in multiple organ systems [7], [8], [9], [10]. In the human prostate, sphere-forming capability enables the selection of a subpopulation of epithelial cells with SC-like traits, including self-renewal and the ability to differentiate into tubular structures when implanted into immunocompromised mice [1], [4]. Previous studies evaluating the antigenic profile of cells capable of forming prostaspheres indicate that they reside within the basal layer of normal prostatic ducts [1], [4], [11], [12]. onsequently, the combination of Trop2 and CD49f^Hi^ expression enables isolation of the basal cell fraction (Trop2^+^CD49f^Hi^), which exclusively forms spheres, regenerates benign tubules, and demonstrates malignant transformation after genetic manipulations [1], [4], [6]. Sphere-forming cells are rare in prostate subpopulations that display luminal profiles (Trop2^+^CD49f^Lo^ or Trop2^+^CD44^−^)[1], [4].

Subdivision of the basal population and enrichment of a sphere-forming and/or tubule-regenerating SC population has yet to be accomplished. However, a functional delineation of the human prostate cellular hierarchy, in addition to basal/luminal profile, could provide more specific insight about the cells of origin for prostate cancer and the pathways utilized by normal SCs that may become corrupted in prostate disease. The aim of this work is to employ in vitro sphere culture and in vivo tissue regeneration assays to interrogate combinations of surface antigens that may further subdivide human prostate epithelial cells and enable functional separation of tubule-initiating SCs from progenitors with more limited capabilities. In this report, we accomplish these goals by incorporating a refined tissue regeneration assay, in which human fetal prostate stroma (hFPS) is utilized to induce tubule formation/differentiation in a fully humanized system. Our results demonstrate that the combination of Epithelial Cell Adhesion Molecule (Epcam), CD44, and CD49f can be used to isolate three distinct populations: (i) a putative prostate SC population that does not form spheres, but induces relatively robust tubule regeneration, (ii) PCs possessing maximal sphere-forming ability, but decreased tubule-initiation capability, and (iii) terminally differentiated LCs that lack both sphere-forming and tissue regenerating potential. The uncoupling of sphere-forming and tubule-initiating functions indicates that human prostate cells with the most potential for niche interaction and tubule development appear to be quiescent in sphere-forming culture conditions.

## Results

### Epcam and CD44 enable separation of prostate cell lineages

**Figure 1:**
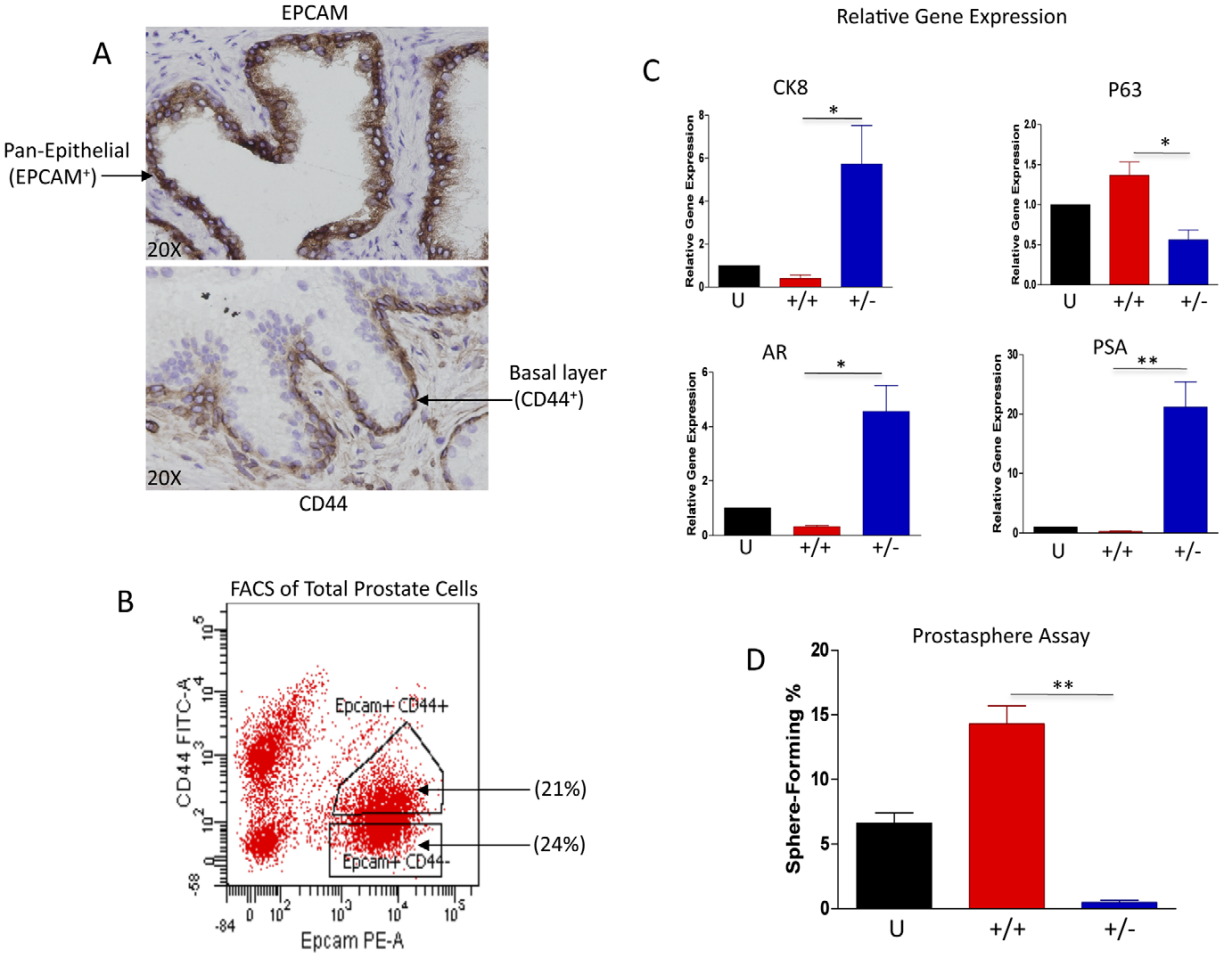
Variation in expression of Epcam and CD44 enables separation of distinct populations of prostate cells from dissociated surgical specimens. A. Immunohistochemical analysis of Epcam and CD44 expression in benign human prostate tissue specimens (20× magnification). B. FACS analysis of Epcam and CD44 expression in total prostate cells isolated from dissociated benign human prostate tissue. Total prostate cells stained with Epcam-PE and CD44-FITC conjugated antibodies prior to FACS analysis. C. Epcam^+^CD44^+^ and Epcam^+^CD44^−^ fractions display basal (P63+) and luminal (CK8+, AR+, PSA+) profiles, respectively. Quantitative RT-PCR reactions were performed in triplicate with a minimum of 3 individual patient specimens. Black columns represent Unfractionated (U) cells, red columns represent Epcam^+^CD44^+^ cells (+/+), and blue columns represent Epcam^+^CD44^−^ cells (+/−). D. Unfractionated prostate epithelial cells isolated from benign prostate tissue specimens or cells fractionated based on Epcam/CD44 expression were evaluated for sphere-forming capability in vitro. 1×10^4^ cells were plated in semi-solid (Matrigel®) cultures. Approximately 14 days after seeding, prostaspheres were quantitated in all wells and the percentage of sphere-forming cells was calculated in each fraction. All experiments were performed in triplicate, using a minimum of three individual patient samples. Statistical analysis was performed using standard one-way ANOVA analysis; P<0.05(*), P<0.01(**).

Epcam/Trop1 is a pan-epithelial antigen that is also expressed on most carcinomas, including prostate cancer [13]. In benign human prostate, immunohistochemical (IHC) staining demonstrates confinement of Epcam expression to epithelial cells that compose prostate ducts and acini (Figure 1A). CD44 is a single pass transmembrane glycoprotein involved in cell-cell matrix adhesion, cell signaling, inflammation, and cell migration ([14]). In the benign human prostate, CD44 marks basal cells and rare neuroendocrine cells [15]. Based on the expression pattern of Epcam and CD44 observed in IHC analysis of benign prostate tubules, it appears that Epcam^+^CD44^+^ cells compose the basal layer, while Epcam^+^CD44^−^ cells appear predominantly luminal (Figure 1A). We hypothesized that fractionating total prostate cells based upon the combination of Epcam and CD44 expression profiles could be a first step in determining antigenic profiles that delineate human prostate cellular hierarchy, by enabling basal and luminal separation. An advantage of both Epcam and CD44 is that conjugated magnetic beads are readily available that enable rapid fractionation of prostate cells without the need for a cell sorter. This may increase the accessibility and feasibility of fractionating surgical specimens. FACS analysis of total prostate epithelial cells using fluorescent antibodies to detect Epcam and CD44 expression demonstrate clear separation of (Epcam^+^) epithelial cells from (Epcam^−^) stromal/blood cells (Figure 1B). Although FACS analysis demonstrates that separation based on CD44 expression is not as distinct as Epcam, both CD44^+^ and CD44^−^ fractions were obtained via cell sorting or magnetic beads separation (Figure 1B).

### Expression of basal- and luminal-specific genes correlates with Epcam/CD44 status

Prostate basal and luminal cells can be distinguished based on marker profile, in addition to architectural organization. The tumor protein p63 is a hallmark indicator of basal cells, which also express relatively low levels of AR and PSA [16], [17]. On the other hand, luminal cells lack p63, but express strong levels of AR, PSA, and cytokeratin 8 (CK8) [18], [19]. In order to confirm enrichment of basal and luminal cells after fractionation based on Epcam/CD44 expression, quantitative RT-PCR analysis was performed on total RNA isolated from fractionated cells with primers targeting basal-specific and luminal-specific genes (Figure 1C). Compared to unfractionated cells and the Epcam^+^CD44^+^ fraction, Epcam^+^CD44^−^ cells demonstrated significantly increased expression of AR, PSA, and CK8 with low relatively low expression of the basal marker, p63. On the other hand, Epcam^+^CD44^+^ cells demonstrated virtually undetectable AR, PSA, and CK8 and enhanced expression of p63. These results are compatible with the known expression profiles of basal and luminal cells and indicate that the combination of Epcam and CD44 can effectively enrich for these lineages [19], [20].

We have previously shown that prostate S/P cells are capable of prostasphere formation in vitro [4]. Additionally, we have found that basal cells are exclusively capable of forming spheres [1]. Therefore sphere-forming capability of Epcam^+^CD44^+^ and Epcam^+^CD44^–^ cell fractions was evaluated in comparison to unfractionated (U) cells. Consistent with previous studies, virtually all of the sphere-forming cells were confined to the basal-enriched Epcam^+^CD44^+^ cell fraction (Figure 1D), and this fraction demonstrated a 3-fold increase in sphere-forming cells compared to unfractionated total prostate cells. This data suggests that Epcam/CD44 fractionation enables a functional segregation of epithelial cell populations, in addition to basal and luminal separation.

### HFPS Supports Prostate Tissue Regeneration Induced by Adult Human Prostate Cells In Vivo

**Figure 2:**
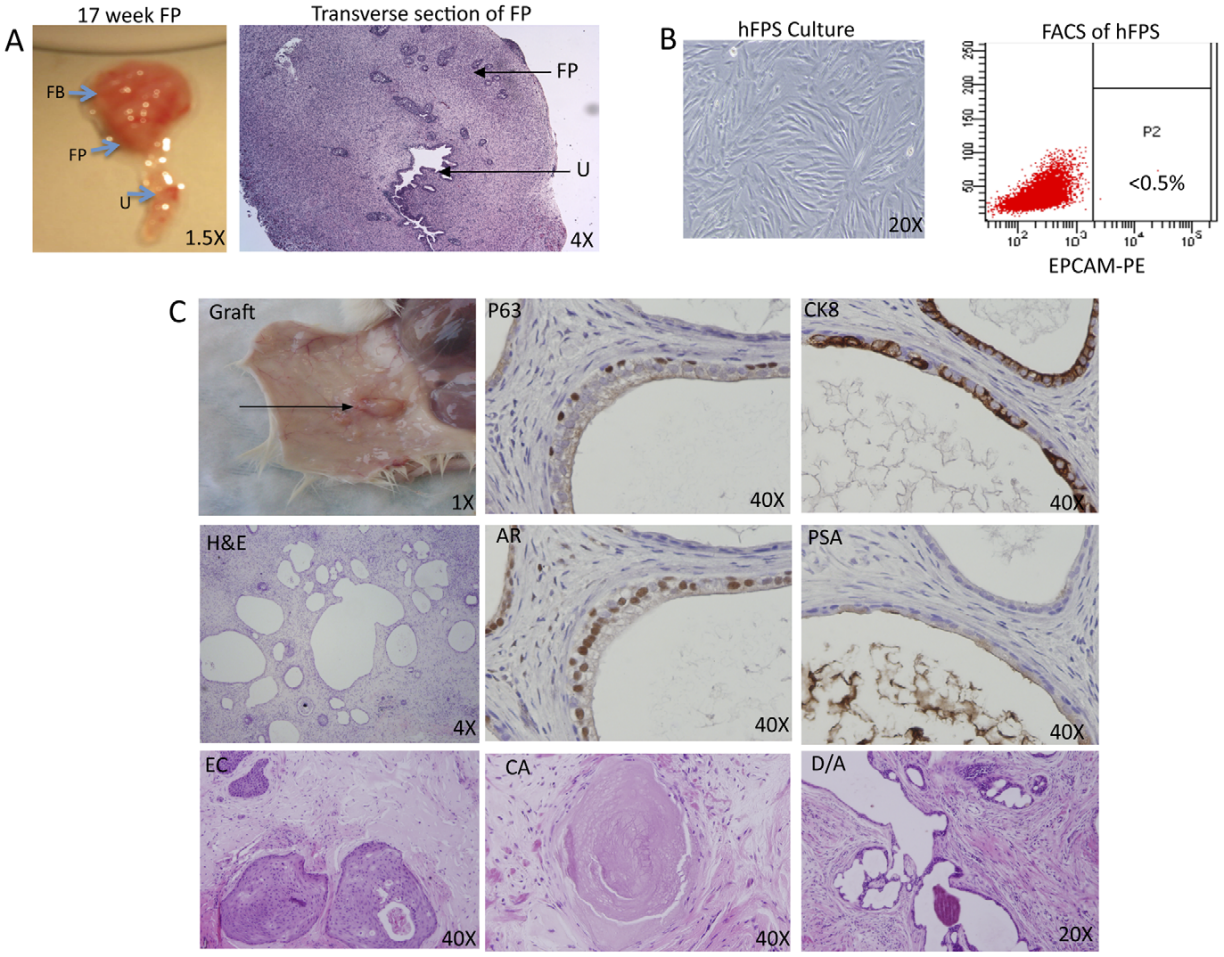
Isolation of human fetal prostate stroma (hFPS) for use in prostate tissue regeneration assays. A. Gross specimen containing 17-week fetal bladder (FB), prostate (FP), and urethra (U) en block with adjacent panel showing transverse hematoxylin and eosin (H&E) stained histological section. Developing prostate glands budding from the prostatic urethra are surrounded by abundant stroma. B. HFPS cells are cultured in DMEM supplemented with FBS. FACS analysis of cultured hFPS cells using antibodies that target Epcam demonstrates lack of (Epcam+) epithelial cell outgrowth. C. Regenerated graft induced by hFPS after recombination with freshly isolated adult human prostate cells and Matrigel®, followed by subcutaneous injection. H&E staining of paraffin-embedded graft demonstrates tubules with a distinct basal layer, containing cells that express tumor protein 63 (P63+) but lack luminal cell marker expression, including Androgen Receptor (AR), cytokeratin 8 (CK8), and Prostate Specific Antigen (PSA). A luminal layer is identified in the majority of outgrowths and contains cells that are P63−, AR+, CK8+, and PSA+. The bottom panel displays the different types of outgrowths identified in recombinant grafts, including epithelial cords (EC), corpora amylacea (CA), and epithelial cords/buds (EC).

We have previously described regeneration of human prostate tissue following implantation of adult prostate cells (or prostaspheres) combined with rat urogenital sinus mesenchyme (rUGSM) and Matrigel® into Non-Obese Diabetic Severely Combined Immunodeficient mice that are Interluekin-2 Receptor Null (SCID-NOD^IL2grNULL^) [4], [6]. In an effort to employ a fully humanized prostate tissue regeneration system, rUGSM was replaced with human prostate stromal cells cultured from dissociated fetal prostate tissue (Figure 2). Histological evaluation of fetal prostate specimens demonstrates abundant stroma surrounding the prostatic urethra with developing epithelial buds/tubules (Figure 2A). FBS-supplemented culture media supported the outgrowth of a nearly pure (Epcam-negative) human fetal stromal cell population (hFPS) that could be passaged continuously for more than 10 generations (Figure 2B and data not shown). Cryopreservation of hFPS, followed by thaw and re-culture enabled further expansion of these cells prior to use in vivo. When hFPS was combined with freshly isolated adult prostate epithelial cells (Figure 2C) or sphere-forming cells (data not shown) and Matrigel®, followed by subcutaneous implantation into SCID-NOD^IL2grNULL^ mice, epithelial cord-like structures formed as early as 6 weeks (data not shown). Fully differentiated ductal/acinar structures with PSA-expressing luminal cells were prominent by 6 months (Figure 2C). Epithelial cords and/or tubular structures failed to form if Matrigel® and hFPS were recombined in the absence of prostate epithelial cells (Figure S1). No differences in tubule development were noted in grafts induced by rUGSM or hFPS (Figure S1). All structures typically identified in benign prostate surgical specimens were present in hFPS regenerated grafts, including epithelial cords, corporal amylacea, and secretion-filled ducts/acini. Layers of epithelial cells expressing basal markers (K5, P63), luminal markers (K8, AR, PSA), or a combination of both were also identified (Figure 2C). HFPS was generated from 6 different fetal specimens and all demonstrated similar growth in culture, FACS profile, and ability to support tubular outgrowth when combined with adult prostate epithelial cells (data not shown).

### Tubule initiating capability is prevalent in the non-sphere-forming Epcam^+^CD44^−^ luminal-enriched cell fraction

Although sphere formation is a common feature of S/Ps, one critical characteristic that prostate SCs must demonstrate is the ability to induce new tubule formation inclusive of ducts/acini composed of both basal and luminal cells. Prostate tissue regeneration assays have been utilized to interrogate the tubule initiation capability of putative S/P populations in mouse and human [4], [21], [22]. In these assays, total or fractionated cells obtained from fetal or adult prostate tissues are combined with supportive stroma (i.e., UGSM) followed by sub-renal implantation as a collagen graft or subcutaneous implantation with Matrigel® into immunocompromised mice. Cell fractions that possess S/P activity induce multi-layered tubular outgrowths with secretions surrounded by stroma. We have previously shown that sphere-forming cells as well as basal cells isolated based on co-expression of Trop2 and high levels of CD49f have an increased ability for tubule initiation compared to luminal (Trop2^+^CD49f^Lo^) cells [1], [4], [6].

**Figure 3:**
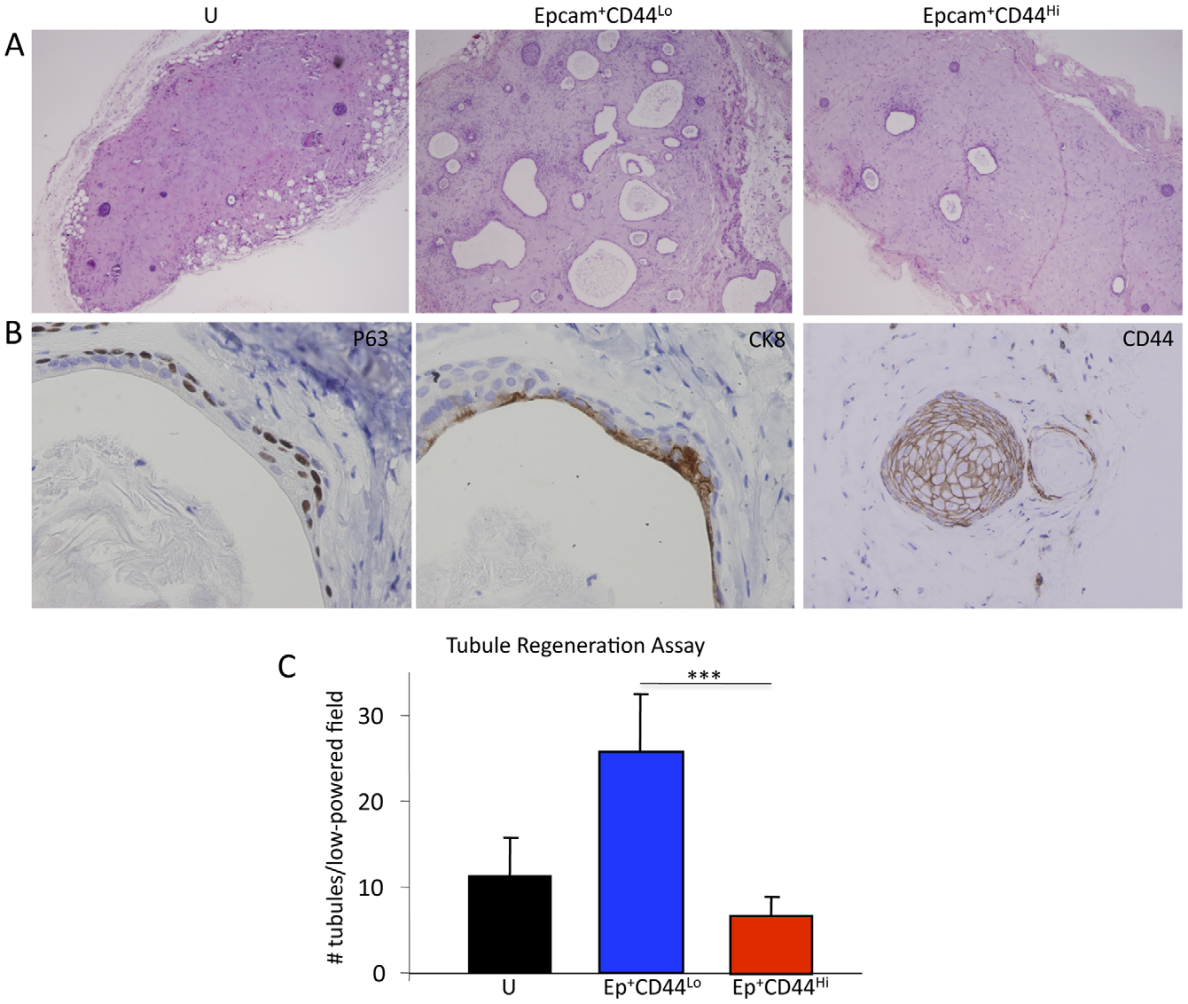
Tubule formation induced by unfractionated and fractionated (Epcam^+^CD44^+^ and Epcam^+^CD44^–^) prostate cells in human prostate tissue regeneration assays. A. H&E stained sections of paraffin-embedded 12-week grafts harvested from SCID-NOD^IL2γrNULL^ mice. Unfractionated (U) total prostate cells or Epcam^+^CD44^+^ and Epcam^+^CD44^−^ cell fractions combined with human fetal prostate stromal cells and Matrigel® were implanted subcutaneously into male SCID-NOD^IL2γrNULL^ mice. Testosterone was supplemented via pellets inserted subcutaneously. B. Example of secretion-filled ducts that display basal (p63 positive) and luminal (CK8 positive) cells induced by Epcam^+^CD44^−^ prostate cell fractions. Tubules and epithelial developed from cords containing CD44^+^ cells also developed from the CD44^–^ cell fraction. C. Comparison of the number of tubular structures identified in unfractionated, Epcam^+^CD44^+^, and Epcam^+^CD44^−^ grafts. After paraffin embedding, sections were made throughout the grafts. The two representative sections containing the highest number of tubules were identified and all tubules present in the low power (4X magnification) field were quantitated. The average numbers of tubules from total grafts obtained from unfractionated or fractionated cells are complied for the graph. Statistical analysis was performed using standard one-way ANOVA analysis; P< 0.001 (***).

In order to investigate the ability of cells fractionated based upon Epcam/CD44 expression to form tubules in vivo, human prostate tissue regeneration was performed. Approximately 1×10^5^ unfractionated cells, Epcam^+^CD44^+^ cells, or Epcam^+^CD44^−^cells were combined with 2×10^5^ hFPS and Matrigel®, followed by subcutaneous implantation into SCID-NOD^IL2grNULL^ mice. Approximately twelve weeks following implantation, grafts were harvested and analyzed for tubule induction via histological analysis of paraffin embedded sections (Figure 3A). A table containing the rate of engraftment of unfractionated and fractionated cells is shown in Figure S2. Tubular structures were identified in grafts that developed from unfractionated cells and in Epcam^+^CD44^+^ recombinant grafts. Surprisingly, the Epcam^+^CD44^−^ luminal enriched/non-sphere-forming fractions yielded the largest number of tubular structures (Figure 3A and 3C). All grafts demonstrated a range of epithelial cord-like structures and more fully developed tubules with secretion-filled lumens (Figure 3A). Immunohistochemical staining confirmed the presence of basal (p63^+^) and luminal (CK8^+^) cells in regenerated tubules (Figure 3B). Although FACS and cytospin examination of fractionated cells confirmed CD44^−^ status (data not shown), CD44^+^ cord-like structures and tubules containing a distinct CD44^+^ basal layer were identified in mature grafts induced by Epcam^+^CD44^−^ cell fractions (Figure 3B). This data suggests that Epcam^+^CD44^−^ cells may be precursors for Epcam^+^CD44^+^ cells found in regenerated tubular structures.

A functional role for a non-sphere-forming/luminal-enriched fraction appeared to contradict prior published results, in which fractionation of luminal cells based on Trop2/CD49f expression displayed no functional capabilities in vitro and in vivo[6]. To investigate this discrepancy, FACS analysis comparing Epcam and Trop2 expression was performed to evaluate co-expression of these surface markers. Indeed, there appeared to be almost complete overlap in expression of Trop2 and Epcam, with virtually all Trop2^+^ cells co-expressing Epcam (Figure S3A). On the contrary, high expression CD49f did not appear to be confined to the CD44+ population, since a fraction of CD44^−^ cells were CD49f^Hi^ (Figure S3B). This result suggests that Epcam^+^CD44^−^ prostate cells may be further subdivided based upon CD49f expression and may explain differential functional capabilities of basal/luminal cell fractions isolated based on Epcam/CD44 profile compared to Trop2/CD49f.

### CD49f enables functional delineation of putative SCs, PCs, and LCs

**Figure 4:**
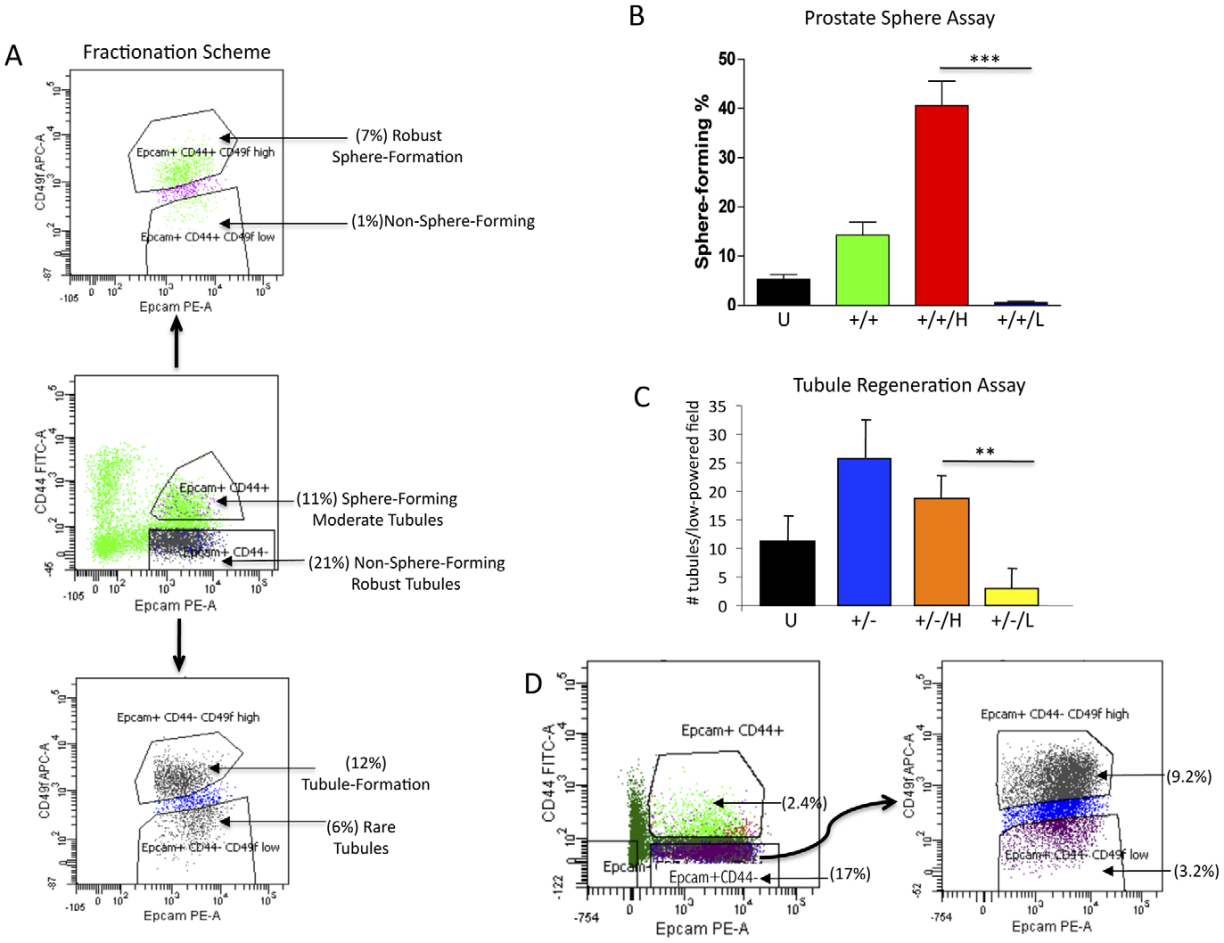
Identification and functional evaluation of CD49f^Hi/Lo^ cells present in Epcam^+^CD44^+^ and Epcam^+^CD44^−^ fractions. A. FACS analysis of Epcam^+^CD44^+^ and Epcam^+^CD44^−^for CD49f^Hi^ expression, with functionally distinct populations annotated. B. Sorting of Epcam^+^CD44^+^ based on CD49f expression followed by sphere analysis in vitro (***P<0.001). Unfractionated (U), Epcam^+^CD44^+^ (+/+), Epcam^+^CD4+^i^CD49f^Hi^ (+/+/H), Epcam^+^CD44^+^CD49f^Lo^ (+/+/L). C. Sorting of Epcam^+^CD44^−^ based on CD49f expression followed by quantification of tubule initiation in vivo. After paraffin embedding, sections were made throughout the grafts. The two representative sections containing the highest number of tubules (4× magnification) were identified and quantitated. The average number of tubules from all the grafts retrieved is represented in the bar graph (**P<0.01). Epcam^+^CD44−CD49f^Hi^ (+/−/H), Epcam^+^CD44^−^CD49f^Lo^ (+/−/L). D. FACS analysis of total cells obtained from three grafts induced by the Epcam^+^CD44^−^CD49f^Hi^ cell fraction. Grafts were mechanically and enzymatically digested to retrieve single cells that were pooled for FACS analysis. Although only highly enriched Epcam^+^CD44^−^CD49f^Hi^ cell fractions were combined with hFPS and Matrigel prior to injection, all of the cell types identified in the original prostate surgical specimens were found in regenerated tissue grafts.

As described above, previous studies indicated that Trop2^+^CD49f^Hi^ basal cells display both sphere forming and tubule regenerating capabilities, compared to the Trop2^+^CD49f^Lo^ luminal cells, which lack these functional capabilities [4], [6]. Given the surprising result that luminal-enriched Epcam^+^CD44^−^ cells display predominant tubule-initiation activity, we investigated whether or not CD49f^Hi^ cells present within this subpopulation may be responsible for tubule initiation in vivo. FACS analysis was performed on total prostate cells after incubation with antibodies targeting Epcam, CD44, and CD49f. Both CD49f^Hi^ and CD49f^Lo^ subpopulations were identified in Epcam^+^CD44^+^ and Epcam^+^CD44^−^ fractions (Figure 4A and S4). Cell sorting enabled isolation of prostate cells based upon Epcam/CD44/CD49 status. Prostasphere culture of Epcam^+^CD44^+^CD49f^Hi^ cells demonstrated enrichment of sphere-forming capability (10-fold over unfractionated cells and 3-fold over Epcam^+^CD44^+^ cells) with 40–50% of cells within this fraction capable of forming spheres (Figure 4B). On the other hand, less than 1% of Epcam^+^CD44^+^CD49f^Lo^ or Epcam^+^CD44^−^CD49f^Hi^ cells were able to form spheres (Figure 4B and data not shown, respectively).

In order to evaluate tubule initiation activity of Epcam^+^CD44^−^ non-sphere-forming cells subdivided by CD49f, in vivo tissue regeneration with hFPS was employed (Figure 4C). Recombinant grafts were retrieved from Epcam^+^CD44^−^CD49f^Hi^ cell fractions containing significantly more tubules than those induced by Epcam^+^CD44^−^CD49f^Lo^ cells (Figure 4C). FACS analysis of dissociated grafts induced by Epcam^+^CD44^−^CD49f^Hi^ cells demonstrated a similar composition of cells (based on Epcam/CD44/CD49f expression) as the original prostate surgical specimen (Figure 4E), indicating that this minority population could induce an intact prostate tissue profile.

As previously described, bright CD49f expression is associated with a basal cell profile, therefore, Epcam^+^CD44^−^CD49f^Hi^ and Epcam^+^CD44^−^CD49f^Lo^ cell fractions were evaluated by RT-PCR analysis to determine if the original Epcam^+^CD44^−^ fraction contained a mix of luminal and basal cells [4], [6], [11]. RNA expression of p63 in association with a lack of AR and PSA indicated that Epcam^+^CD44^−^CD49f^Hi^ cells possessed a basal profile, while Epcam^+^CD44^−^CD49f^Lo^ cells exhibited a luminal profile, demonstrated by significant AR and PSA expression (Figure S5). This contrasting expression profile of Epcam^+^CD44^−^CD49f^Hi^ cells compared to RT-PCR analysis of Epcam^+^CD44^−^ cells (in which fractionation with CD49f was not performed), indicates that the luminal expression profile observed with Epcam^+^CD44^−^ cell fractions was likely due to a masking effect by true luminal cells (Epcam^+^CD44^−^CD49f^Lo^) that co-segregated with the non-sphere-forming basal subpopulation (Epcam^+^CD44^−^CD49f^Hi^). Taken together, these results suggest that the human prostate basal cell population can be divided into populations with enriched sphere-forming activity (Epcam^+^CD44^+^CD49f^Hi^) or tubule-initiating activity (Epcam^+^CD44^−^CD49f^Hi^).

## Discussion

Identifying functionally distinct populations of prostate epithelial cells could provide new insights about the cells of origin for human prostate cancer, by determining which cells within the hierarchy are susceptible to malignant transformation. Additionally, the mechanisms employed by normal prostate SCs that enable interaction with the niche and initiation of tubule development could lead to therapeutic approaches that interfere with similar pathways exploited by cancer cells or contributing to the development of benign prostatic hypertrophy (BPH). revious studies investigating human prostate S/P cells isolated from benign tissues have indicated that both general epithelial and basal antigens (Trop2, CD44, alpha_2_beta_1_-integrin^Hi^, alpha_6_-integrin^Hi^ (CD49f)) are expressed [1], [2], [4]. In these studies, the ability to form self-renewing prostaspheres coincides with the potential to induce fully differentiated prostate tubules in vivo. In the current study, subpopulations of prostate basal cells with robust sphere-forming capability are distinguished from those with optimal tubule initiating capability based on specific antigenic profiles. Prostate epithelial cells with an increased potential to induce tubules inclusive of basal and luminal cell layers (putative SCs) are incapable of forming prostaspheres in vitro. On the other hand, highly proliferative sphere-forming cells (putative PCs) appear to have more limited potential for tubule initiation. This study is the first to functionally separate prostate epithelial cells based upon sphere-forming versus tubule initiating capabilities.

Combinations of antigens that subdivide the basal population and functionally distinguish prostate SCs from PCs have not been reported, with the exception of CD133, a rare surface marker found on less than 1% of basal cells [2]. A recent report regarding α_2_β_1_^Hi^CD133^+^ cells indicated that these cells were incapable of forming spheres, but readily formed proliferative monolayer cultures [23]. Additional studies have demonstrated acinar-like outgrowths induced by α_2_β_1_^Hi^CD133^+^ cells, in vivo [2]. This combined data suggests that CD133^+^ cells are non-sphere-forming, but possess SC traits of self-renewal and differentiation capability, similar to the Epcam^+^CD44^−^CD49f^Hi^ population reported here. In previous studies, we have also reported that CD133 expression did not enrich for sphere-forming cells [4]. However, given the surprising new finding of increased tubule formation induced by the non sphere-forming, Epcam^+^CD44^−^CD49f^Hi^ cell fraction, analyzing concomitant expression of CD133 (and other putative SC markers) within this subset, including further fractionation and functional analysis, should be considered.

In previous studies of prostate S/P cells, Trop2, which has an almost identical pattern of expression as Epcam (Trop1) within prostate epithelial cells, was utilized to separate prostate epithelial from stromal and blood cells [1]. One advantage of using Epcam, as an alternative to Trop2, is stable and/or highly expressed Epcam is detected in most adenocarcinomas, as well as metastases, malignant effusions, and cancer stem cells [24]. Confirming the presence of Epcam within the human prostate S/P population may lead to investigations of therapeutic agents targeting Epcam and evaluation of specific effects on prostate SC and PC activity [24], [25].

In the current study, CD44 expression appears to determine whether Epcam^+^ prostate epithelial cells will form robust spheres (CD44^+^) or remain quiescent in vitro, but induce robust tubule formation in vivo (CD44^−^). In the neural system, it is a well-recognized limitation that quiescent neural SCs cannot be isolated using the neurosphere assay [26]. Additionally, it is emphasized that sphere-formation and self-renewal is a trait possessed by both SCs and PCs. In the current study, the antigenic profile of cells with the highest prostate sphere-forming capability is Epcam^+^CD44^−^CD49f^Hi^. However, sphere-forming cells marked by Epcam^+^CD44^+^ expression can form tubules in vivo, but at a statistically significant lower rate than non-sphere-forming Epcam^+^CD44^−^ cells. Since previous in vivo studies clearly demonstrate that CD49f^Hi^ is required for prostate tubule formation, we hypothesized that the Epcam^+^CD44^−^CD49f^Hi^ antigenic profile designates non-sphere-forming cells capable of tubule regeneration in vivo. Indeed, this antigenic profile was confirmed in our study to represent a subpopulation of prostate basal cells with relatively robust tubule-initiating capability (compared to Epcam^+^CD44^−^CD49f^Hi^ luminal cells) [6]. In contrast to our sphere results, sub-fractionation of Epcam^+^CD44^−^ cells with increased tubule initiation capability did not appear to further enrich for this activity. One factor that may have contributed to this observation is the fact that FACS sorting with three markers requires longer sort time, which could impact the long-term viability of these cells that is required for in vivo grafting. Despite enrichment with the more refined cell fraction, our results clearly demonstrate an advantage in tubule formation capability compared to luminal Epcam^+^CD44^−^CD49f^Lo^ cells. Consequently, three distinct populations of prostate epithelial cells are revealed, including subdivided basal (Epcam^+^CD44^+^CD49f^Hi^ and Epcam^+^CD44^−^CD49f^Hi^) and luminal (Epcam^+^CD44^−^CD49f^Lo^) fractions.

Bona fide SCs should be capable of residing in the quiescent state and become activated to differentiate and form new tubules as needed. With asymmetric cell division, progenitor daughter cells develop with less potential to induce new tubules. In the current study, although some sphere-forming cells retain the potential to induce new tubules, the proportion is far less than the in vitro quiescent Epcam^+^CD44^−^population. This result implies that prostaspheres contain both SCs and rapidly proliferating progenitors (possible transit-amplifying cells), resulting in an overall decreased potential to induce tubules compared to non-sphere-forming SCs. Hence, Epcam^+^CD44^Hi^CD49f^Hi^ cells may be further along the developmental pathway and suggests a hierarchy of prostate epithelial cells.

Although the sphere-forming assays indicate that our putative SCs are quiescent, further studies are needed to evaluate this trait. It has been suggested that sphere-formation is an indicator of self-renewal, yet we have found that the non-sphere-forming (Epcam^+^CD44^−^CD49f^Hi^) cells are capable of inducing differentiated tubules and regenerated grafts that include the full spectrum of prostate cells found in original surgical specimens, including putative SCs. This data indicates that in addition to differentiation and niche interaction capabilities, the putative SCs are self-renewing (despite the inability to form spheres).

Taken together, our results suggest that Epcam^+^CD44^Lo^CD49f^Hi^ cells are non-sphere-forming SCs that may be activated to form tubules when exposed to inductive stroma cells in vivo. Lack of CD44 expression distinguishes non-sphere-forming SCs from the more proliferative state of the CD44^+^ population, which may contain an increased proportion of PCs with limited induction potential, relative to tubule-initiating SCs. Support for CD44 as a proliferative marker exists. The majority of primary prostate epithelial cells (transient amplifying cells) that grow as a monolayer, in vitro, express CD44 [27], [28], [29]. Examination of human prostate cancer cell lines and xenografts indicate that the CD44^+^ population is more proliferative, clonogenic, tumorigenic, and metastatic than CD44^−^ cells[30], [31], [32].

Future studies that may yield more insight into prostate SC/PC characteristics and function include gene expression array analysis comparing Epcam^+^CD44^−^CD49f^Hi^ and Epcam^+^CD44^+^CD49f^Hi^ cells. Such efforts could reveal novel antigens and genetic pathways that are unique to each subpopulation. Additionally, genetic manipulation of benign prostate cell fractions based on Epcam/CD44/CD49f expression, followed by in vivo regeneration may suggest mechanisms of tumorigenesis or benign proliferation (BPH) at different developmental stages.

## Methods

### Tissue Digestion and Cell Dissociation

Human prostate tissue was obtained via a research protocol that was approved by the Office for the Protection of Research Subjects at UCLA and the Greater Los Angeles VA Medical Center. Informed written consent was obtained on all participants where identifying information was included. In cases where no identifying information was included and tissue was acquired in an anonymous fashion at UCLA, an approved Institutional Review Board protocol with written consent was not required by Office for the Protection of Research Subjects. Adjacent tissue specimens were snap frozen in liquid nitrogen or fixed in formalin and paraffin-embedded for histological analysis. Frozen sections were immediately examined by a genitourinary pathologist and cancer foci encircled. Fresh tissue specimens were matched with the frozen section slides to enable macrodissection of benign tissue away from tumor nodules. Typically, 2–10 grams of fresh tissue was allocated for research studies. Tissue specimens were then mechanically and enzymatically digested as previously described [16]. Dissociated tissue containing single cells and organoids was sequentially filtered through 100-µm and 40-µm cell strainer, and then passed repeatedly through a 23-gauge needle, in order to generate a single cell suspension. Cells were counted with a hemocytometer and resuspended in RPMI supplemented with 10% FBS prior to cell sorting or plating in prostasphere cultures. Approximately 1–2 million viable cells per gram of fresh tissue were routinely obtained.

### Magnetic activated cell sorting(MACS)

Miltenyi auto MACS® was used to separate Epcam^+^CD44^+^ and Epcam^+^CD44^−^ prostate epithelial cells. For Epcam^+^ cell separation, single cell suspensions obtained from freshly dissociated prostate tissue were stained with anti-human Epcam-PE antibody (Miltenyi Biotech), followed by incubation with anti-PE Multisort Microbeads (Miltenyi Biotech). Stained cells were separated through autoMACS (Miltenyi Biotech) with Mode POSSEL (Positive Selection). Positive fraction was collected as Epcam^+^ cells and microbeads were removed using Multisort Release Reagents (Miltenyi Biotech). Cells were then stained with CD44 microbeads before separation through auto MACS® separator with POSSEL, with collection of positive (Epcam^+^CD44^+^) and negative (Epcam^+^CD44^−^) fractions. The negative fraction was separated further with Mode DEPLETES (Depletion in sensitive mode. The Epcam^+^CD44^+^ and Epcam^+^CD44^−^ cells were stained with anti-human CD44-PE-Cy-7 (eBioscience) and analyzed by FACS to evaluate the purity of sorted cells.

### Fluorescence-activated cell sorting (FACS)

Prostate cells were suspended in PBS, 2 mM EDTA,0.5%BSA and stained with antibody for 15 minutes at 4°C. Fluorescence-activated cell sorting and analysis were performed on a BD Special Order FACS Aria II system and Diva v6.1.1 (BD Biosciences). Live single cells were gated based on scatter properties and analyzed for their surface marker expression. Cells were sorted and collected at 4°C using 100um nozzle and 23psi. Antibodies used for FACS include Epcam-PE (Miltenyi Biotech), CD44-FITC (ebioscience), and CD49f-APC (BioLegend).

### In vitro prostasphere assay

Prostate cells were counted and re-suspended in 50∶50 Matrigel: PrEGM with a concentration of 5×10^3^ cells/80microliters. This Matrigel/cellular suspension was plated at the edge of the well on 12-well plates and allowed to solidify by incubation at 37°C for 30 minutes. One milliliter of defined sphere media was then added to each well and plates were replaced in 37°C incubator, as previously described [4]. Quantitation of prostaspheres was performed approximately 10–14 days after plating.

### Tissue acquisition, isolation and culture of fetal prostate cells

Human fetal prostate tissue was acquired from 16–17 week specimens in accordance with federal and state guidelines. Adjacent prostate tissue was snap frozen in liquid Nitrogen or fixed in formalin and paraffin-embedded to evaluate anatomy and glandular architecture. The remainder of the tissue was mechanically and enzymatically digested as described (13). Dissociated prostate cell suspensions were sequentially filtered through 100-micron and 40-micron filters, and then passed through a 23-gauge needle. Cells were counted with a hemocytometer and resuspended in RPMI supplemented with 10% fetal bovine serum (FBS), penicillin/streptomycin (Mediatech Inc.), and Methyltrienolone R1881 (Sigma) for culture in vitro. After 3 passages, cells were analyzed via FACS to confirm purity of stromal cells (See below). HFBS is cryopreserved and thawed as needed for use in recombination assays.

### In vivo tissue regeneration

In vivo tissue experiments were performed in male SCID-NOD^IL2grNULL^ mice in accordance with protocol number 2007-189-11A, approved by the Animal Research Committee within the Office for the Protection of Research Subjects at UCLA. Mice (6–8 weeks old) were subjected to subcutaneous injections of prostate epithelial cells. Approximately 1×10^5^ epithelial cells were combined with 2×10^5^ primary human fetal prostate stroma cells (hFPS). The epithelial and stromal cells were then suspended in 50 microliters 50∶50 Matrigel®: PrEGM. Subcutaneous implantation of time-release testosterone pellets (Innovative Research of America) was simultaneously performed at the time of graft implantation. Subcutaneous nodules at the site of injection were removed after approximately 12 weeks of the implantation and frozen/paraffin-embedded sections were generated for immunohistochemical analysis. Fresh hFPS cells were cultured in RPMI supplemented with 10% FBS and R1881 (Sigma) and passaged three times prior to use in tissue regeneration assays.

### Immunohistochemistry of tissue sections

Prostate tissue was paraffin embedded as previously described [33]. Four-micron thick sections of frozen or paraffin embedded tissue were deparaffinized with xylene and rehydrated through a descending series of ethanol washes as described [4]. Antigen retrieval and standard immunoperoxidase procedures were used in combination with primary antibodies, including CK5, CK8 (Convane), p63, androgen receptor (AR), Prostate Specific Antigen (PSA) (Santa Cruz), and CD44 (Abcam).

### Real time RT-PCR Analysis

RNA was extracted using Qiagen RNAeasy Micro Kit, following the manufacturer's instructions. The concentration and purity of total RNA was assessed spectrophotometrically at 260 and 280 nm. CDNA was generated from total RNA (up to 5 µg) using SuperScript III First-Strand Synthesis Kit (Invitrogen). For quantitative Real-time PCR, a Bio-Rad CFX Multicolor Real-time PCR detection system was employed, using the SYBR®-Green Supermix (Bio-Rad Laboratories). Real-time PCR primer pairs for CK8, PSA, AR and p63 were purchased from SABiosciences Corporation. The PCR reaction conditions included an initial step at 95°C for 3 min, followed by 40 cycles at 95°C for 15 s (Melt) and 60°C for 45 s (Anneal/Extend). Detection of PCR products was accomplished by measuring the emitting fluorescence at the end of each reaction step (reaction cycles). Threshold cycle corresponds with the cycle number required to detect a fluorescence signal above the threshold. Calculations were performed by Bio-Rad IQ5 software provided by the manufacturer. Gene expression analysis was performed using the comparative method.

## Supporting Information

Figure S1 **Comparison of prostate tissue grafts induced by rUGSM and hFPS.** Total adult prostate cells (5×10^5^) isolated from fresh benign surgical specimens were combined with either rUGSM or hFPS (1×10^6^ cells). Grafts were retrieved approximately 12 weeks following subcutaneous injection into SCID-NOD^IL2γrNULL^ mice. H&E staining of paraffin-embedded sections demonstrated similar composition of tubular structures within grafts, including ductal/acini structures, corpora amylacea, and epithelial cords. Similar to previous studies with rUGSM, grafts that formed from hFPS without additive adult prostate epithelial cells (PCs) did not contain any tubular structures. All grafts with tubules (T) were found to have prominent vasculature (BV) throughout (Right panel).
(TIF)

Figure S2 **Table depicting number of patient samples utilized for implants and grafts retrieved.** A total of 29 implants yielded 20 grafts with tubules for comparative analysis (69% engraftment rate).
(TIF)

Figure S3 **Epcam (Trop1) and Trop2 demonstrate overlapping expression in human prostate cells, while CD49f and CD44 demonstrate disparate expression.** A. Total prostate cells were co-stained with antibodies recognizing Epcam and Trop2 and subjected to FACS analysis. The majority of Epcam^+^ cells co-expressed Trop2. B. Total prostate cells were co-stained with antibodies recognizing CD44 and CD49f. A population of CD49f^Hi^ cells were identified that appear to be CD44^−^, suggesting that a proportion of Epcam^+^CD44^−^cells may co-express CD49f.
(TIF)

Figure S4 **FACS analysis of individual patient surgical specimens for Epcam/CD44/CD49f.** Four patient specimens (A–D) are shown for comparative analysis of populations retrieved. After mechanical and enzymatic digestion, single cell suspensions are stained with antibodies targeting Epcam, CD44, and CD49f. High and low CD44-expressing populations of Epcam+ cells are gated and analyzed for CD49f expression. High and low CD49f-expressing cells are then isolated for functional analysis.
(TIF)

Figure S5 **Quantitative RT-PCR demonstrates Epcam^+^CD44^−^CD49f^Hi^ cell fractions have a basal profile (p63^+^AR^Lo^PSA^−^), while Epcam^+^CD44^−^CD49f^Lo^ cells display a luminal profile (p63^Lo^AR^Hi^PSA^+^).** Primers targeting p63, AR, and PSA were used in fractionated cells to compare expression relative to unfractionated cells (U). Epcam^+^CD44^−^CD49f^Hi^ (+/−/H), Epcam^+^CD44^−^CD49f^Lo^ (+/−/L). Statistical analysis was performed using standard one-way ANOVA analysis. P<0.05(*), P<0.01(**).
(TIF)
