# Supplementary figures and images for: Epcam, CD44, and CD49f Distinguish Sphere-Forming Human Prostate Basal Cells from a Subpopulation with Predominant Tubule Initiation Capability
Source: PLoS One. 2012 Apr 13;7(4):e34219. doi: 10.1371/journal.pone.0034219 (PMC3326009; doi:10.1371/journal.pone.0034219)

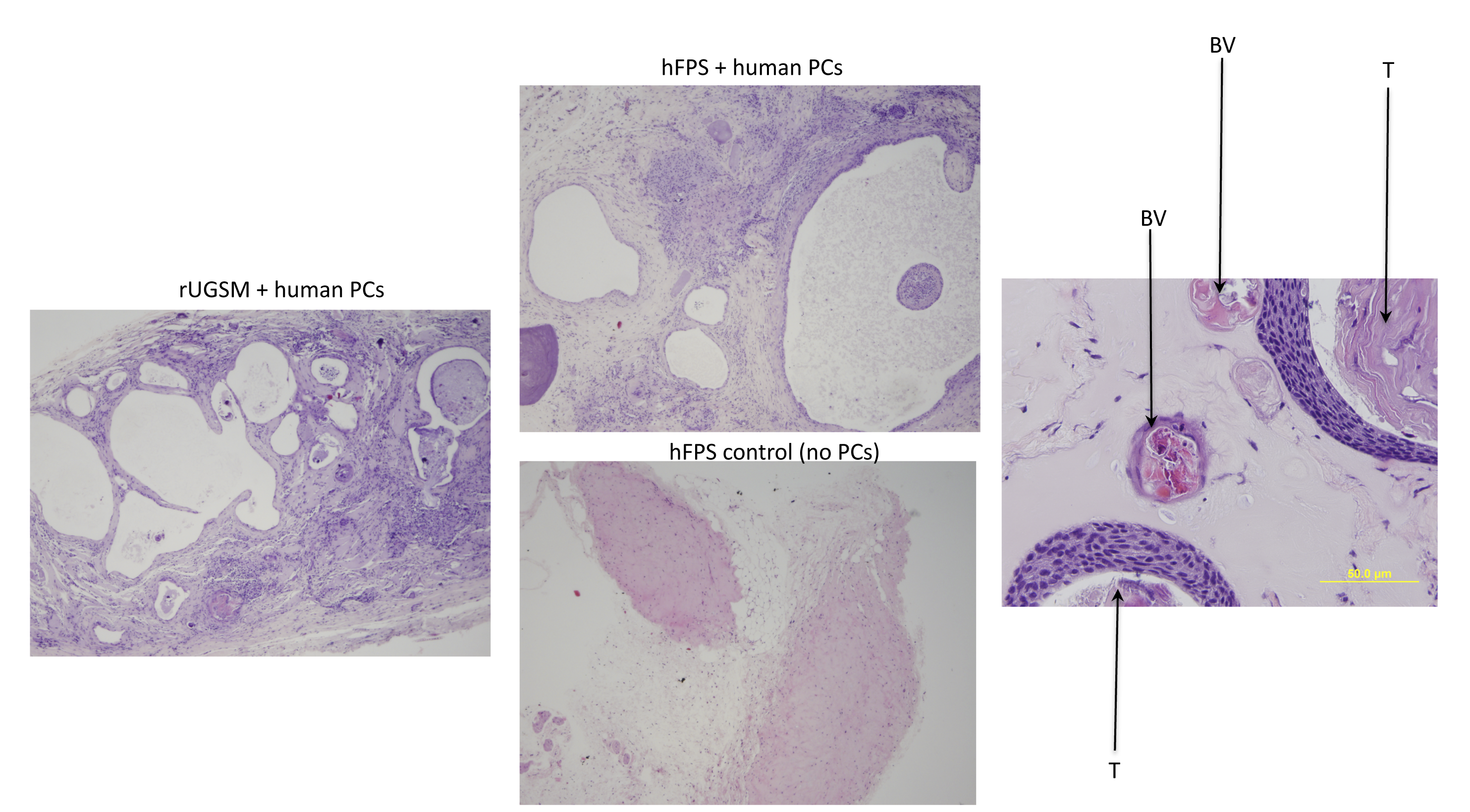

Supplement: Figure S1 — Comparison of prostate tissue grafts induced by rUGSM and hFPS. Total adult prostate cells (5×10^5^) isolated from fresh benign surgical specimens were combined with either rUGSM or hFPS (1×10^6^ cells). Grafts were retrieved approximately 12 weeks following subcutaneous injection into SCID-NODIL2γrNULL mice. H&E staining of paraffin-embedded sections demonstrated similar composition of tubular structures within grafts, including ductal/acini structures, corpora amylacea, and epithelial cords. Similar to previous studies with rUGSM, grafts that formed from hFPS without additive adult prostate epithelial cells (PCs) did not contain any tubular structures. All grafts with tubules (T) were found to have prominent vasculature (BV) throughout (Right panel). (TIF) [file pone.0034219.s001.tif]

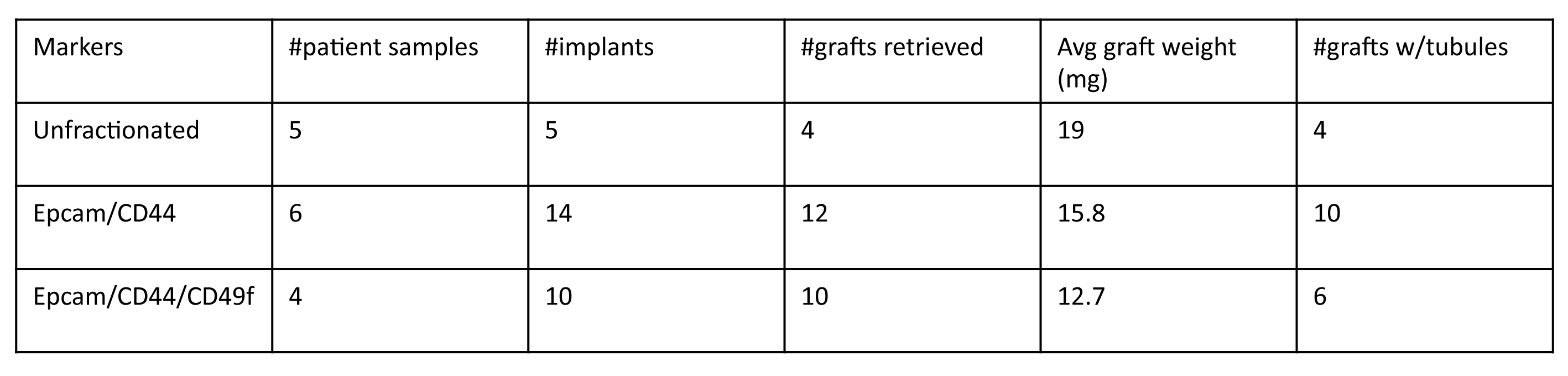

Supplement: Figure S2 — Table depicting number of patient samples utilized for implants and grafts retrieved. A total of 29 implants yielded 20 grafts with tubules for comparative analysis (69% engraftment rate). (TIF) [file pone.0034219.s002.tif]

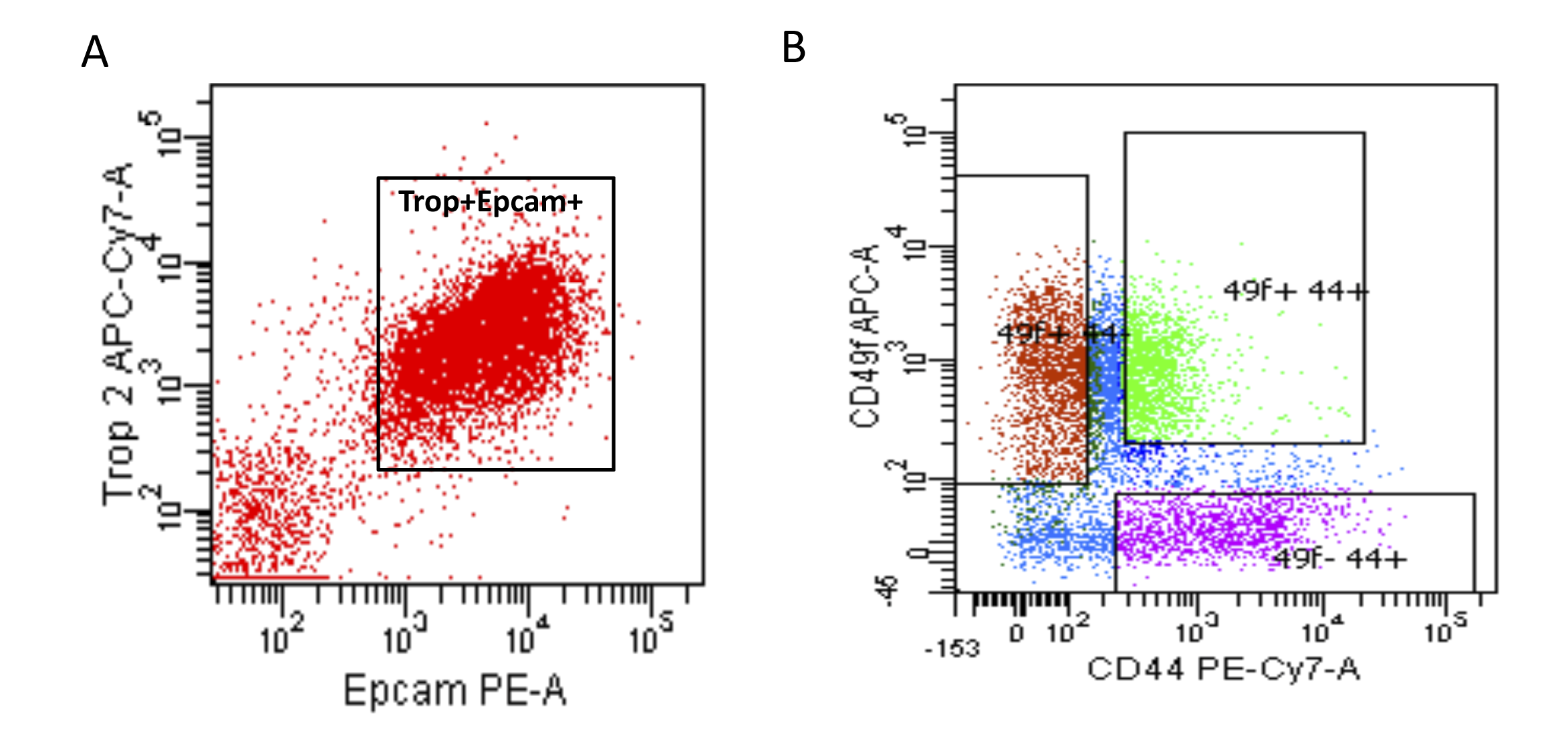

Supplement: Figure S3 — Epcam (Trop1) and Trop2 demonstrate overlapping expression in human prostate cells, while CD49f and CD44 demonstrate disparate expression. A. Total prostate cells were co-stained with antibodies recognizing Epcam and Trop2 and subjected to FACS analysis. The majority of Epcam+ cells co-expressed Trop2. B. Total prostate cells were co-stained with antibodies recognizing CD44 and CD49f. A population of CD49fHi cells were identified that appear to be CD44−, suggesting that a proportion of Epcam+CD44−cells may co-express CD49f. (TIF) [file pone.0034219.s003.tif]

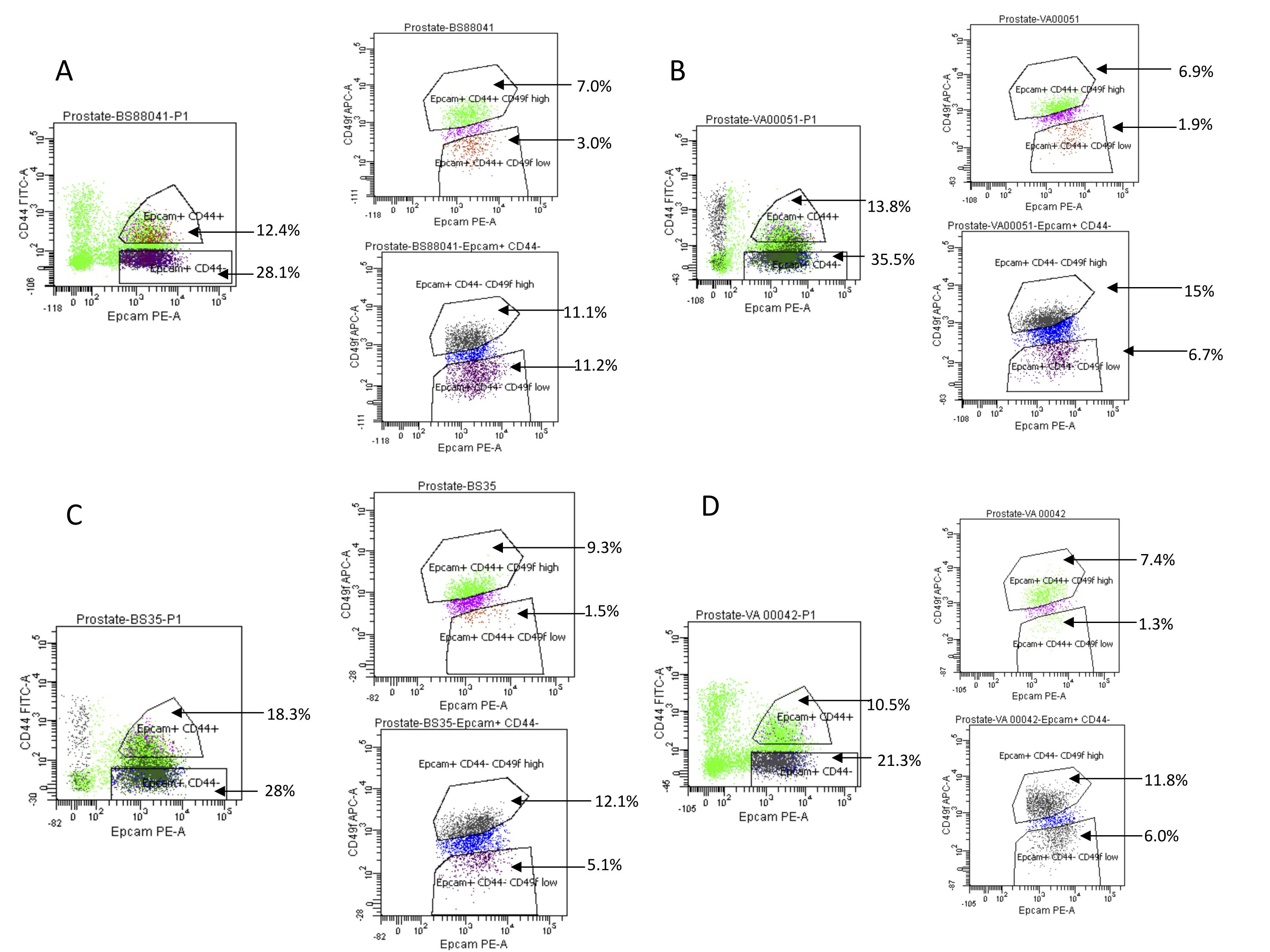

Supplement: Figure S4 — FACS analysis of individual patient surgical specimens for Epcam/CD44/CD49f. Four patient specimens (A–D) are shown for comparative analysis of populations retrieved. After mechanical and enzymatic digestion, single cell suspensions are stained with antibodies targeting Epcam, CD44, and CD49f. High and low CD44-expressing populations of Epcam+ cells are gated and analyzed for CD49f expression. High and low CD49f-expressing cells are then isolated for functional analysis. (TIF) [file pone.0034219.s004.tif]

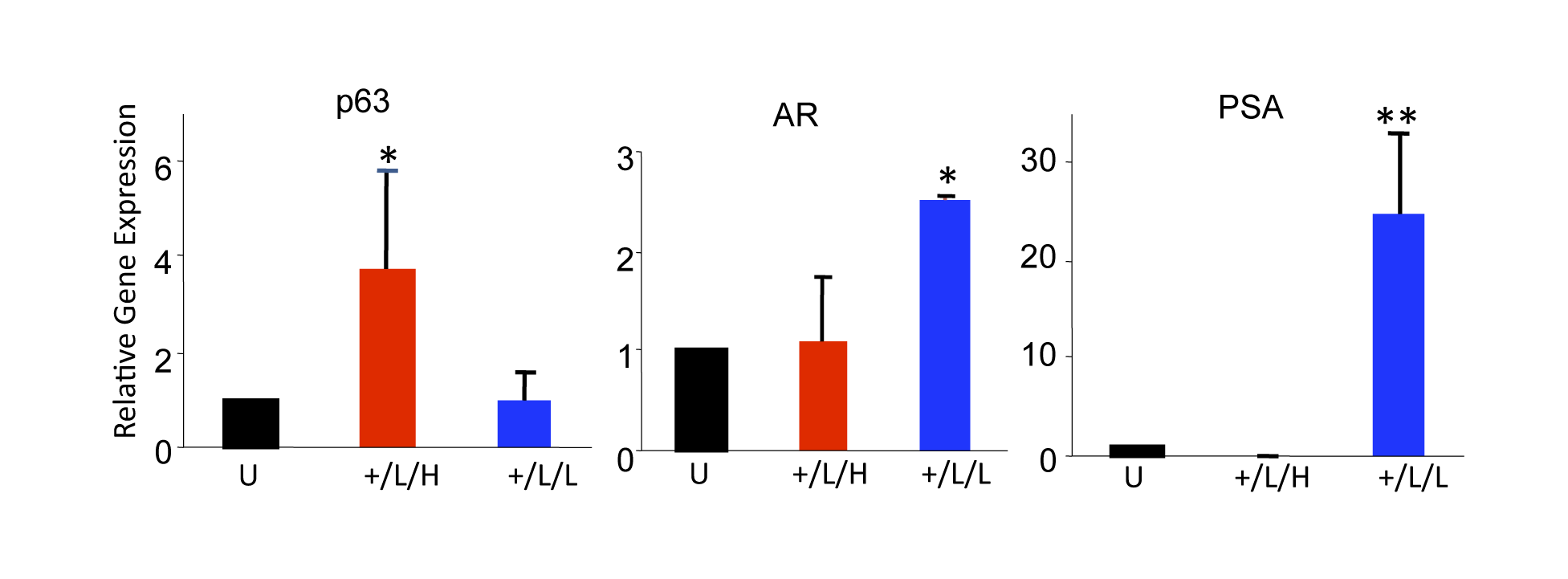

Supplement: Figure S5 — Quantitative RT-PCR demonstrates Epcam+CD44−CD49fHi cell fractions have a basal profile (p63+ARLoPSA−), while Epcam+CD44−CD49fLo cells display a luminal profile (p63LoARHiPSA+). Primers targeting p63, AR, and PSA were used in fractionated cells to compare expression relative to unfractionated cells (U). Epcam+CD44−CD49fHi (+/−/H), Epcam+CD44−CD49fLo (+/−/L). Statistical analysis was performed using standard one-way ANOVA analysis. P<0.05(*), P<0.01(**). (TIF) [file pone.0034219.s005.tif]
